# Dual inhibition of EGFR and VEGFR pathways in combination with irradiation: antitumour supra-additive effects on human head and neck cancer xenografts

**DOI:** 10.1038/sj.bjc.6603791

**Published:** 2007-06-26

**Authors:** A Bozec, P Formento, S Lassalle, C Lippens, P Hofman, G Milano

**Affiliations:** 1 Oncopharmacology Unit, Centre Antoine-Lacassagne, Nice, France; 2 Department of Pathology, University Hospital, Nice, France

**Keywords:** AZD2171, gefitinib, radiotherapy, head and neck cancer, antiangiogenic, tyrosine kinase inhibitor

## Abstract

The aim of this study was to investigate the effects of combining antiangiogenic treatment, epidermal growth factor receptor (EGFR) targeting and irradiation (RT). We evaluated AZD2171, a highly potent, orally active, vascular endothelial growth factor (VEGF) signalling inhibitor, gefitinib, an EGFR tyrosine kinase inhibitor and RT. The antitumour efficacy of these treatments, administered alone and in combination for 2 weeks, was assessed in a VEGF-secreting human head and neck tumour cell line, CAL33 that highly expresses EGFR, established as xenografts (250 mm^3^) in nude mice. The median time to reach a tumour volume of 1000 mm^3^ was significantly increased for AZD2171 or gefitinib alone compared with the control. Greater inhibition of tumour growth was seen with the combination of AZD2171+gefitinib compared with either drug alone, and the triple combination compared with either AZD2171+gefitinib or RT alone. The intensity of endothelial cell staining was slightly reduced by each agent given alone, and markedly diminished by the double or triple combination. The triple combination almost completely abolished cell proliferation. The marked RT-induced enhancement in the DNA-repair enzyme ERCC1 expression was totally abolished by the triple combination. This observation could help to explain the supra-additive antitumour effect produced by this combination and could provide a basis for future innovative clinical trials.

Agents that target epidermal growth factor receptor (EGFR) potentially exert antitumour effects by inhibiting tumour cell proliferation and survival, as well as reducing the secretion of proangiogenic growth factors such as vascular endothelial growth factor (VEGF) and fibroblast growth factor that stimulate tumour neoangiogenesis (Perrotte *et al*, 1999; Woodburn, 1999; Ciardiello *et al*, 2001). It has been reported that EGFR targeting in tumours may also modulate the migration and formation of tube-like structures of vascular endothelial cells (Hirata *et al*, 2002). More recently, we have shown the presence of a gefitinib-sensitive functional EGFR pathway in an immortalised microvascular endothelial cell line of human origin (Bozec *et al*, 2005). Thus, in addition to direct effects on tumour cells, EGFR-targeting drugs may also impart an indirect antitumour effect through antiangiogenic activity. An optimal antiangiogenic strategy may, therefore, be to combine an EGFR signalling inhibitor with an agent that selectively targets VEGF-dependent signalling.

The novel indole-ether quinazoline, AZD2171, is a highly potent (IC_50_< 1 nM) ATP-competitive inhibitor of recombinant VEGF receptor-2 (VEGFR-2) tyrosine kinase *in vitro* (Wedge *et al*, 2005). AZD2171 also shows potent activity *vs* VEGFR-1 (IC_50_=5 nM) and VEGFR-3 (IC_50_⩽3 nM). In human umbilical vein endothelial cells, AZD2171 inhibited VEGF-stimulated proliferation and VEGFR-2 phosphorylation with IC_50_ values of 0.4 and 0.5 nM, respectively. In a fibroblast/endothelial cell coculture model of vessel sprouting, AZD2171 reduced vessel area, length and branching at sub-nanomolar concentrations. The growth of established human tumour xenografts in athymic mice was dose-dependently inhibited by chronic administration of AZD2171. Combining AZD2171 and gefitinib could potentially provide inhibitory effects on both endothelial and tumour cells. Recent preclinical studies suggest that radiotherapy (RT) in combination with antiangiogenic/vasculature-targeting agents may enhance the therapeutic ratio of ionising radiation alone (Wachsberger *et al*, 2005; Cao *et al*, 2006; Williams *et al*, 2007). Thus, the combination of AZD2171 and gefitinib with RT could also be interesting to investigate.

Tumour vasculature is a key target in the treatment of solid tumours, particularly in head and neck cancer (Le and Giaccia, 2003). In the present study, the antitumour activity of AZD2171, in combination with gefitinib and RT on a number of cellular and molecular markers in human head and neck tumour xenografts (CAL33), was investigated.

## MATERIALS AND METHODS

### Chemicals

AZD2171 and gefitinib were kindly provided by AstraZeneca, Macclesfield, Great Britain. Working solutions were prepared as follows: AZD2171 (375 mg l^−1^) and gefitinib (7.5 g l^−1^) were suspended in 0.9% NaCl, 0.01% Tween 80. For both drugs, the concentrations were adjusted so as to include the daily dose in 0.2 ml of drug suspension. Dulbecco's modified Eagle's medium (DMEM), penicillin, streptomycin and glutamine were purchased from Whittaker (Verviers, Belgium). Foetal bovine serum (FBS) was obtained from Dutscher (Brumath, France).

### Cell lines

CAL33, a cell line of human head and neck origin, was obtained from our institution (Centre Antoine-Lacassagne, Nice, France). This cell line exhibits high EGFR levels (33 794±624 fmol mg^−1^ protein high-affinity sites determined by ligand binding assay; Dassonville *et al*, 1993) and produces VEGF (C Onesto, CNRS-UMR6543, personal communication).

The cell line was maintained as monolayer culture in DMEM supplemented with 10% FBS v*/*v, 2 mM glutamic acid, 50 000 U l^−1^ penicillin and 80 *μ*M streptomycin in a humidified incubator (Sanyo, Osaka, Japan) at 37°C in an atmosphere containing 8% CO_2_. Batches of 15 × 10^6^ cells were frozen in FBS supplemented with 5% dimethyl sulphoxide (v*/*v) in advance for injection into mice. Shortly before injection, cells were thawed and suspended in Ringer lactate.

### Mice

Animal experiments were performed in accordance with the regulations of our institution's ethics commission and with the United Kingdom Co-ordinating Committee on Cancer Research guidelines (Workman *et al*, 1998). Six-week-old male NMRI nude mice were purchased from Janvier laboratories (Le Genet sur Isle, France) and received s.c. inoculation in the right flank of 2 × 10^6^ cells suspended in 100 *μ*l of Ringer lactate (*n*=10 per treatment condition). There were five animals per cage with food and water *ad libitum*; following tumour cells injection, animal weight and tumour growth were monitored once a week. Animals were killed at the end of experiment by cervical disruption; as no signs of suffering appeared during the experiment (submitted attitude, weight loss, prostration, vocalisation), no animal has to be killed before the end of experiment.

### Treatment

Mice bearing well-established CAL33 tumours (mean tumour volume/treatment group ∼250 mm^3^) were treated each week with vehicle alone (controls), AZD2171 (2.5 mg kg^−1^ every day 0.2 ml p.o.), gefitinib (50 mg kg^−1^ day^−1^, 5 days per week, 0.2 ml p.o.) and RT (6 Gy day^−1^, 3 days per week, 2 h after drugs administration) for 2 weeks. When coadministered, AZD2171 and gefitinib were given simultaneously. The dose of gefitinib and AZD2171 were chosen according to preliminary experiments so that each drug given alone exerts only partial effects on tumour growth.

Radiotherapy was performed with *γ*-rays on tumour only, using a ^60^Co unit, the animals being maintained and biologically isolated from ambient air during RT with a straitjacket made of Saran wrap film.

The effect of AZD2171 and gefitinib alone or in combination with RT on tumour growth was evaluated. The effects of the treatments were calculated, as described previously (Prewett *et al*, 2002). Evaluation of the effects on tumour growth consisted in measuring, for all groups, the mean tumour volume at the end of the observation period for the controls (day 30), when tumours in this group reached the average volume of 2500 mm^3^ (maximal ethically acceptable volume). Fractional tumour volume (FTV) for each treatment group was calculated as the ratio between the mean tumour volumes of treated and untreated animals. This was performed for treatment a (FTVa), for treatment b (FTVb) and for treatment a+b (FTV a+b). The expected FTV for the a+b combination was defined as FTVa observed × FTVb observed. The ratio FTVa+b expected/FTVa+b observed was the combination ratio (CR). If CR>1, there are supra-additive effects and if CR<1 infra-additive ones. Strictly additive effects were observed if CR=1. Another way to compare treatment effects was to calculate the time necessary for the tumours to reach a volume of 1000 mm^3^ (log-rank test).

### Tumour analysis

Tumour length and width was measured weekly using a calliper rule. Tumour volume was calculated as *π*/6 × length × width^2^ until animal killing. Animals were killed by spinal cord dislocation and tumours were subsequently removed surgically. Half of the tumour was directly frozen in liquid nitrogen for protein analysis and the other half fixed in paraformaldehyde overnight for analysis of the microvessel marker, von Willebrand factor (vWF; Dako, polyclonal antibody ref.: A0082), and the proliferation marker, Ki67 (Dako, Trappes, France, monoclonal antibody ref.: M7240, MIB-1), using immunohistochemistry after tumour tissue from the experimental groups was assembled into microtissue arrays (Simon *et al*, 2004). The analysis of vWF concerned both vessel loss and reduction in vessel area. The analysis of Ki67 took into account both the intensity of labelling and proportion of labelled cells.

The final score was the result of the examination of three fields per tumour, and between four and five tumours were investigated for each treatment group. One hundred × 10 magnification images of regions of high vascular density within the tumour were analysed in order to quantify tumour angiogenesis (vWF). Staining intensities were scored as: 1=slight; 2=medium; and 3=strong. A subgroup (*n*=5) was killed on day 23 (the last day of treatment) and tumours collected, while the remaining animals (*n*=5) were killed in each group, once animals in the group reached a tumour volume of 2500 mm^3^. Approximately, these times were reached on day 30 for control and gefitinib, on day 37 for AZD2171 and AZD2171+gefitinib, on day 50 for RT, and on day 60 for AZD2171+gefitinib+RT treatment groups.

Frozen tumours were pulverised in a liquid nitrogen-cooled Thermovac pulveriser. The resulting powders were homogenised in 10 volumes of a 10 mM Tris-HCl buffer pH 7.4, containing 1 mM EDTA, 0.5 mM dithiothreitol, 10 mM sodium molybdate, phosphatase inhibitor cocktail 2 with a dilution 1/100 and protease inhibition cocktail 2 with a dilution 1/100, both from Sigma (Saint Quentin Fallavier, France). The homogenates were centrifuged for 1 h at 105 000 **g** (+4°C) and the supernatants (cytosols) were used for protein determination by immunoblotting. Total protein content was measured using the bicinchoninic acid assay.

Human VEGF secreted by CAL33 xenografted tumours was determined in tumour cytosol by ELISA using DVE00 (Quantikine, R&D systems, Lille, France). Epidermal growth factor receptor had been previously determined by the I^125^-EGF-binding method followed by Scatchard-plot analysis (Dassonville *et al*, 1993).

The EGFR and VEGFR signalling pathway markers phospho-ERK1/2, PTEN and phospho-AKT, the apoptosis-related markers Bax/Bcl2 ratio, the proliferation-related marker p27 and the DNA repair-related marker ERCC1 were determined by Western blot (detailed technical conditions are summarised in Table 1).

Two sets of tumours have been analysed with different purposes. One set (five animals per treatment group) was collected at the end of treatment period (day 23) in order to compare the effects of the different treatments on a panel of molecular markers. The other set of tumour-bearing animals was maintained to assess tumour regrowth after the end of treatment period. Animals were killed when mean tumour volume of each treatment group reached 2500 mm^3^. These tumours were used to detect what molecular parameters could be modified in these tumours escaping to treatments. The first set of tumours was analysed for vWF (endothelial cell-specific marker) and Ki67 (proliferative capacity of tumour cells) by immunohistochemistry, for the EGFR and VEGFR signalling pathway markers such as phospho-ERK1/2 and phospho-AKT, for the apoptosis-related markers Bax/Bcl2 ratio, the proliferation-related markers p21 and p27 and the DNA repair-related marker ERCC1 (essentially on tumour cells as they largely outnumber endothelial cells in tumours) by Western blot normalised by Raf as a loading control, and VEGF (human, secreted by tumour cells only) by ELISA.

The second set of tumours was analysed for phospho-ERK1/2, phospho-AKT and PTEN expression by Western blot also normalised by Raf as a loading control.

The bands in the Western blots were quantified using the Chemi Doc imager from Bio-Rad, Marnes-La-Coquette, France.

### Statistical analyses

Comparison of tumour growth between different treatment groups was performed by Kaplan–Meier-type analysis with the log-rank statistical test. The effects of treatment on vWF and Ki67 were evaluated using the non-parametric ANOVA (Kruskal–Wallis test). The differences between treatment groups for molecular factors (VEGF, phospho-ERK1/2, PTEN, phospho-AKT, Bax/Bcl2, ERCC1) were examined using the Mann–Whitney test.

## RESULTS

### Effects of AZD2171 in combination with gefitinib and radiation on tumour growth

**Figure 1:**
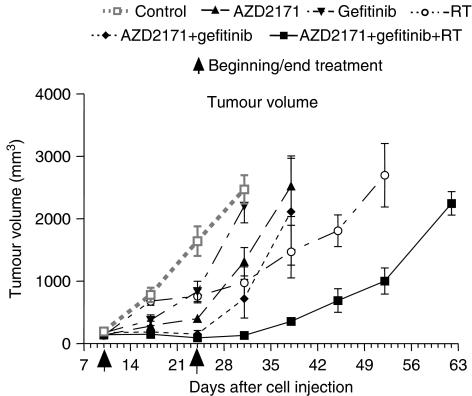
Effects of AZD2171 (2.5 mg kg^−1^, every day during 2 weeks), gefitinib (50 mg kg^−1^, 5 days per week during 2 weeks), RT (6 Gy, three times per week, every 2 days during 2 weeks, 2 h after drugs) or a combination on growth of CAL33 head and neck tumours (mean tumour volume±s.d., *n*=10 per treatment condition until day 24, then *n*=5 per treatment group except for RT where *n*=4).

The effect of the different treatments on tumour growth is shown in Figure 1. Tumour growth was inhibited by AZD2171 or gefitinib given alone. These antitumour effects were rapidly established but disappeared after treatment cessation. In contrast, the antitumour effects of RT were only seen after 1 week of treatment and persisted over a longer period. Combination treatment with AZD2171+gefitinib produced a greater inhibition of tumour growth than either treatment alone and led to a growth arrest. Tumour regrowth occurred after the end of treatment in all groups with either drugs alone or when combining AZD2171 with gefitinib.

We then considered the AZD2171–gefitinib combination as a single-drug treatment and examined the effects of its combination associated with RT. The triple combination (AZD2171+gefitinib+RT) produced a greater antitumour effect than either RT or the AZD2171–gefitinib combination. In comparison to RT or to the AZD2171–gefitinib combination, the triple combination prolonged the antitumour effects after treatment discontinued. Following the treatment period with the triple combination, growth arrest lasted for 1 week, followed during 3 weeks by a growth paralleling that of the RT-treated tumours and by a rapid growth during the last 10 days.

Antitumour effects for AZD2171+gefitinib in combination were supra-additive (CR=1.6) as were those for the triple combination of both drugs administered with RT (CR=2). These CR values (>1) are consistent with tumour growth inhibition observed with each regimen.

**Figure 2:**
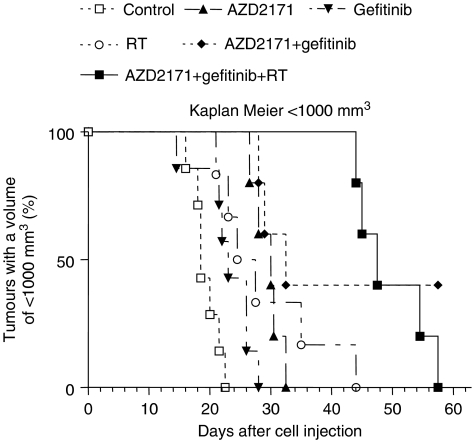
Respective time delay to reach a tumour volume of 1000 mm^3^ for the different treatment groups (seven mice for control and gefitinib, six mice for RT and five mice for all other treatment groups). Of note, two tumours never reached the 1000 mm^3^ volume (these tumours were purely necrotic but still present at the end of the experiment).

The median time to reach a tumour volume of 1000 mm^3^ was significantly increased for AZD2171 or gefitinib alone compared with the control (*P*=0.001). There was also a significant difference between the combination of AZD2171+gefitinib and either drug alone (*P*=0.006) and between the triple combination and AZD2171+gefitinib (*P*=0.0001) or RT alone (*P*<0.0001; Figure 2).

**Figure 3:**
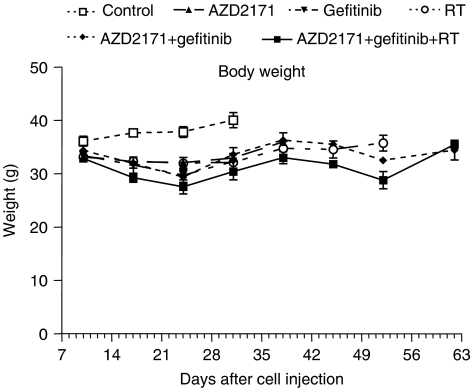
Profile of body weight *vs* time among the different treatment groups (mean weight±s.d., *n*=10 per treatment condition until day 24, *n*=5 thereafter).

Body weights were measured as surrogate markers of treatment tolerance with a slight body weight loss in all treatment groups compared to the control group, thus suggesting that treatments were well tolerated (Figure 3).

### Effects of treatments on molecular parameters

**Figure 4:**
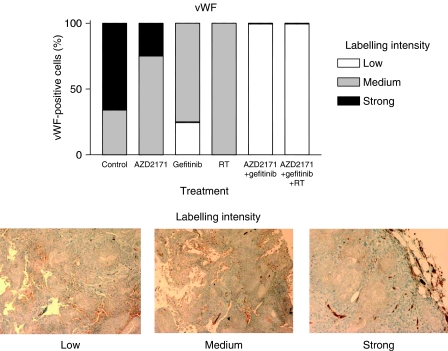
Impact of the different treatments on day 24 at the end of the treatment period for the different treatments on vWF (endothelial cell marker), four microscope fields observed on four tumours (conditions AZD2171, gefitinib, AZD2171–gefitinib), five tumours (conditions RT, AZD2171–gefitinib–RT), six tumours (control). Magnification × 20 for labelling intensity.

In the first set of tumours (collected at the end of treatment period), the intensity of vWF staining was slightly reduced by each agent given alone, and markedly diminished by the double or triple combination (*P*<0.0001 for triple association *vs* control; Figure 4).

**Figure 5:**
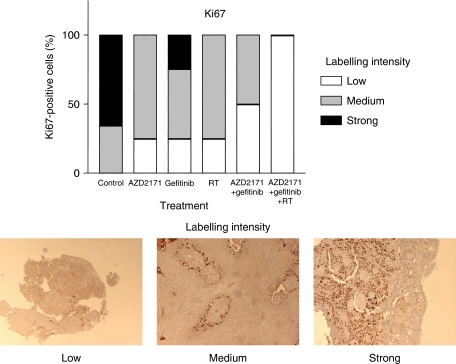
Impact of the different treatments on Ki67 staining (proliferation marker) on day 24 at the end of the treatment period for the different treatments (four microscope fields observed) on four tumours (conditions AZD2171, gefitinib, AZD2171–gefitinib), five tumours (conditions RT, AZD2171–gefitinib–RT), six tumours (control). Magnification for labelling intensity: × 4 for low and × 20 for medium and strong.

The triple combination almost completely abolished proliferation in tumour cells as shown by the decrease in Ki67 labelling intensity (*P*<0.001 for triple association *vs* control; Figure 5). The effects on apoptosis-related factors Bax/Bcl2 ratio were inconsistent (data not shown). No significant effect on phospho-ERK1/2 and on phospho-AKT was observed with any of the treatment groups (data not shown).

**Figure 6:**
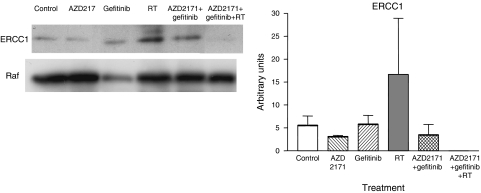
Effect of the different treatments on the expression of ERCC1 on day 24 at the end of the treatment period for the different treatments (mean expression±s.d.), three tumours for control and AZD2171–gefitinib–RT and four tumours for all other treatment conditions. A typical example of Western blot analysis is given. The results were normalised *vs* Raf taken as a loading control.

The marked RT-induced enhancement in ERCC1 expression was totally abolished by the triple combination (*P*=0.04 for the triple combination *vs* RT; Figure 6). This result could help to explain the supra-additive antitumour effect produced by this combination.

**Figure 7:**
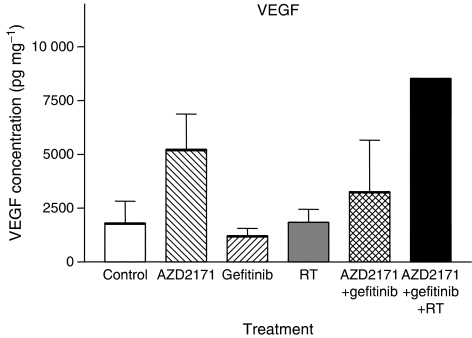
Human VEGF tumour concentration (pg mg^−1^ protein) on day 24 at the end of the treatment period for the different treatments (mean concentration±s.d.), three tumours for controls and AZD2171, four tumours for conditions with gefitinib, RT and AZD2171–gefitinib; the triple combination could be analysed on one tumour only, due to the very small size of the tumours reflecting the efficiency of the combined treatment.

Compared with VEGF levels in the control tumours, gefitinib alone tended to decrease VEGF concentration (*P*=0.157), whereas AZD2171 alone enhanced levels of VEGF in the tumour (*P*=0.02 *vs* control). The combination AZD2171+gefitinib as well as RT alone had no effect on VEGF levels, whereas the triple combination was associated with an increase in VEGF (Figure 7).

**Figure 8:**
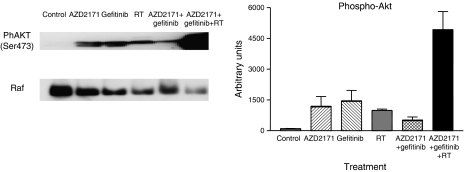
Phospho-AKT expression for tumours in regrowth following different treatments. Tumours collected when mean tumour volume in each group reached 2500 mm^3^ (mean±s.d., three tumours for each treatment condition). A typical example of Western blot analysis is given. The results were normalised *vs* Raf taken as a loading control.

**Figure 9:**
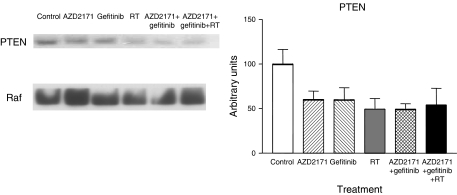
PTEN expression for tumours in regrowth following the different treatments. Tumours collected at the end of follow-up when mean tumour volume in each group reached 2500 mm^3^ (mean±s.d., three tumours for each treatment condition). A typical example of Western blot analysis is given. The results were normalised *vs* Raf taken as a loading control.

**Figure 10:**
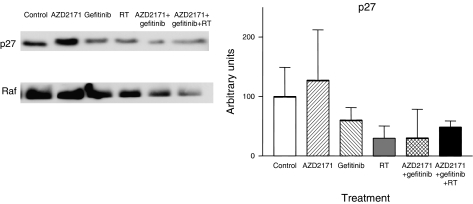
p27 expression for tumours in regrowth following the different treatments. Tumours collected when mean tumour volume in each group reached 2500 mm^3^ (mean±s.d., three tumours for each treatment condition). A typical example of Western blot analysis is given. The results were normalised *vs* Raf taken as a loading control.

In addition to measurements of tumour factors at the end of treatment, there were molecular investigations performed in the second set of tumours (obtained at the end of follow-up when tumours had regrown). All tumours were collected at a mean volume of 2500 mm^3^. There was a variable diminution of phospho-ERK1/2 expression as compared to the control for all treatment conditions (data not shown). All the treatment groups showed an increase in phospho-AKT expression with the triple association showing the largest increase (*P*<0.0001; Figure 8). All treatment groups showed a marked decrease in PTEN expression, the phosphorylase, which negatively controls the PI3-Akt pathway (reaching statistical significance for RT and gefitinib+AZD2171 combination only, *P*=0.04 and 0.03, respectively; Figure 9). These results corroborate the effects observed on phospho-AKT. In addition, all treatments except gefitinib alone showed a marked decrease in the expression of the cell-cycle regulating protein p27 (*P*=0.001; Figure 10). Results with p21 were less consistent (data not shown).

## DISCUSSION

Several preclinical studies have examined the antitumour activity of inhibitors of EGFR and antiangiogenic agents in combination and have demonstrated at least additive, if not synergistic, effects (Ciardiello *et al*, 2000; Jung *et al*, 2002; Herbst *et al*, 2003). These encouraging data have led to the initiation of clinical studies in lung cancer, evaluating the combination of erlotinib, an EGFR tyrosine kinase inhibitor, with bevacizumab, an anti-VEGF antibody (Herbst *et al*, 2005). Previous experimental studies showed potential beneficial antitumour effects when combining antiangiogenic agents with RT (Wachsberger *et al*, 2005, Nieder *et al*, 2006), resulting in at least additive effects on tumour growth delay despite different radiation schedules, drugs and doses and combination regimens. Clinical research in this field is ongoing but additional preclinical studies are needed to further evaluate drug combinations, including the targeting of EGFR and VEGF signalling pathways in association with RT. The present study was designed in a similar way to work published by Raben *et al* (2004) who combined gefitinib with vandetanib (a vascular-targeting agent) and RT. The authors reported that the triple association induced the greatest effect on tumour growth and angiogenesis.

The present data indicate that the double combination, the concomitant application of the VEGF signalling inhibitor, AZD2171, with gefitinib produced more than additive effects with a CR value of 1.6, and that the triple combination, the concomitant application of the VEGF signalling inhibitor, AZD2171–gefitinib association with RT, produced the highest supra-additive antitumour effects with a CR value of 2. Importantly, this triple combination maintained a longer growth delay in comparison either to AZD2171–gefitinib double combination or to RT. The exploration of several tumour markers sustained the antitumour effects, which were observed. Among them, it was interesting to note that the antitumour efficacy of the triple combination could be attributed both to a direct impact on tumour cell proliferation (Ki67 with a marked decrease in labelling) and on the endothelial cell network of the tumour (vWF staining). A previous experimental study by our group was performed on an immortalised microvascular endothelial cell line of human origin (Bozec *et al*, 2005). This cell line was exposed to a combination, including gefitinib, with ZM317450 a tyrosine kinase inhibitor against VEGFR-2 and RT. A marked synergistic interaction for cytotoxic effect was found with this triple combination only. These data concur well with the present results showing a decrease of the number of endothelial cells, as indicated by vWF staining, by combining AZD2171 with gefitinib and RT compared to untreated controls and either monotherapy. It was not within the scope of the present study to examine the effects of the respective associations between single drugs and RT. The AZD2171–gefitinib combination was taken as a whole when combined with RT.

Blood plasma levels of VEGF are significantly increased by VEGFR-2 blockade in mice and were proposed as a surrogate marker for VEGFR-2 targeted therapy in the clinical situation (Jain *et al*, 2006). Sunitinib, a tyrosine kinase inhibitor of VEGFRs, was shown to increase plasma levels of VEGF in treated patients (Faivre *et al*, 2006a). The present data indicate that AZD2171, a highly potent VEGFR-2 inhibitor, was able to increase VEGF tumour expression. The strict tumour origin of the measured VEGF was based on the fact that the measured VEGF was of human origin. This observation confirms that changes in VEGF levels under treatment may be the reflect of modifications occurring in tumour VEGF expression.

One of the molecular explanations for the supra-additive effects on tumour growth presently observed may lie in the changes in tumour expression of the DNA repair protein ERCC1. As expected, ERCC1 expression was markedly enhanced by RT treatment alone. When combined with AZD2171 and gefitinib, this RT-dependent induction of ERCC1 was totally abrogated (Figure 6).

PTEN inactivation and constitutive activation of AKT are well-defined genetic alterations in the initiation and progression of tumours (Vivanco and Sawyers, 2002). AKT is a crucial survival kinase, and interfering with AKT expression is an attractive strategy to control tumour progression (Goswami *et al*, 2006). Interestingly, the present data show that tumour regrowth was accompanied by marked changes in tumour expression of PTEN and phospho-AKT. PTEN was found to be significantly decreased. Conversely, phospho-AKT expression was enhanced as compared to controls. This was particularly true in tumours having shown the highest response under treatment (with the triple combination), AKT is one of the main mTOR-related messengers (Smolewski, 2006) and the current development of mTOR inhibitors as anticancer drugs is very promising (Faivre *et al*, 2006b). It is suggested that to add mTOR targeting as an additional sequence of treatment following the presently studied combination could maintain prolonged antitumour effects over time. Overall, the present data highlight the interesting antitumour effects of the triple combination with gefitinib, AZD2171 and RT. This strategy could serve as a basis for future innovative clinical trials.

## Figures and Tables

**Table 1:** Technical conditions of Western blot analyses

**Antibody**	**% Acrylamide**	**Dilution**	**Source**	**Supplier**
PTEN	12	1/1000 TBS 5% milk	Mouse	Pharmingen
ERCC1	12	1/500 TBS 5% milk	Mouse	Neomarkers
Phospho-AKT	12	1/1000 TBS 5% BSA	Rabbit	Ozyme
P27	12	1/1000 TBS 5% milk	Rabbit	Pharmingen
Bax	12	1/1000 TBS 5% milk	Rabbit	Upstate
Bcl2	12	1/1000 TBS 5% milk	Rabbit	Pharmingen
Phospho-ERK1/2	12	1/5000 TBS 5% milk	Mouse	Sigma

BSA=bovine serum albumin; TBS=tris-buffered saline.
